# Modeling heading and path perception from optic flow in the case of independently moving objects

**DOI:** 10.3389/fnbeh.2013.00023

**Published:** 2013-04-01

**Authors:** Florian Raudies, Heiko Neumann

**Affiliations:** ^1^Center for Computational Neuroscience and Neural Technology, Boston UniversityBoston, MA, USA; ^2^Center of Excellence for Learning in Education, Science, and Technology, Boston UniversityBoston, MA, USA; ^3^Institute for Neural Information Processing, University of UlmUlm, Germany

**Keywords:** optic flow, self-motion, independently moving object, 3D motion models, kinetic contours

## Abstract

Humans are usually accurate when estimating heading or path from optic flow, even in the presence of independently moving objects (IMOs) in an otherwise rigid scene. To invoke significant biases in perceived heading, IMOs have to be large and obscure the focus of expansion (FOE) in the image plane, which is the point of approach. For the estimation of path during curvilinear self-motion no significant biases were found in the presence of IMOs. What makes humans robust in their estimation of heading or path using optic flow? We derive analytical models of optic flow for linear and curvilinear self-motion using geometric scene models. Heading biases of a linear least squares method, which builds upon these analytical models, are large, larger than those reported for humans. This motivated us to study segmentation cues that are available from optic flow. We derive models of accretion/deletion, expansion/contraction, acceleration/deceleration, local spatial curvature, and local temporal curvature, to be used as cues to segment an IMO from the background. Integrating these segmentation cues into our method of estimating heading or path now explains human psychophysical data and extends, as well as unifies, previous investigations. Our analysis suggests that various cues available from optic flow help to segment IMOs and, thus, make humans' heading and path perception robust in the presence of such IMOs.

## Introduction

Optic flow, the apparent change of structured patterns of light on the retina during head, eye, or body motions, contains cues about these motions and the viewed environment. For instance, optic flow contains information about the 3D linear velocity and the 3D rotational velocities, e.g., the yaw, pitch, and roll velocity, of the eyeball (Longuet-Higgins and Prazdny, 1980). In addition, optic flow includes motion parallax cues that, e.g., provide a relative-depth order between foreground objects and background (Helmholtz, 1925; Gibson et al., 1995). Self-motion in a rigid environment results in optic flow that is entirely defined by 3D linear and 3D rotational velocity parameters and depths as seen from the observer's point of view. In practical situations the environment is non-rigid, e.g., other people walk within the visual field. Flow generated by these moving people is inconsistent with the parameters of self-motion. Instead, this flow is a superposition of the self-motion and other people's motion. Therefore, our goal is to analyze segmentation cues and their possible effect on the estimation of self-motion by integration of flow from only rigid parts in the environment.

Our work is motivated by three studies. Two studies measure a heading bias for linear motion toward fronto-parallel planes where one plane moves independently, the independently moving object (IMO), and another serves as rigid background (Warren and Saunders, 1995; Royden and Hildreth, 1996). The third study measures accuracy of path perception for curvilinear motion above a ground plane where IMOs are modeled as cubes (Fajen and Kim, 2002; see Warren and Mestre, 1991 for path perception without IMOs). In the first two studies heading biases are present either if the IMO obscures the focus of expansion (FOE) of the background motion or if the IMO's path crosses the observer's path. The FOE is a point in the image plane that is introduced by linear motion and appears at the intersection of the heading vector, or linear velocity vector, with the image plane. The third study did not find any significant differences in path perception whether IMO's had been present or not while changing their position, path, or degree of transparency. These seemingly contradictory results from these studies raise the question: what are the differences in task and configuration in the experiments, and to what extent do these explain the heading bias or absence of error in path perception? Furthermore, we ask: what are the mechanisms that enable humans to robustly perceive heading or path under these circumstances?

**Figure 1:**
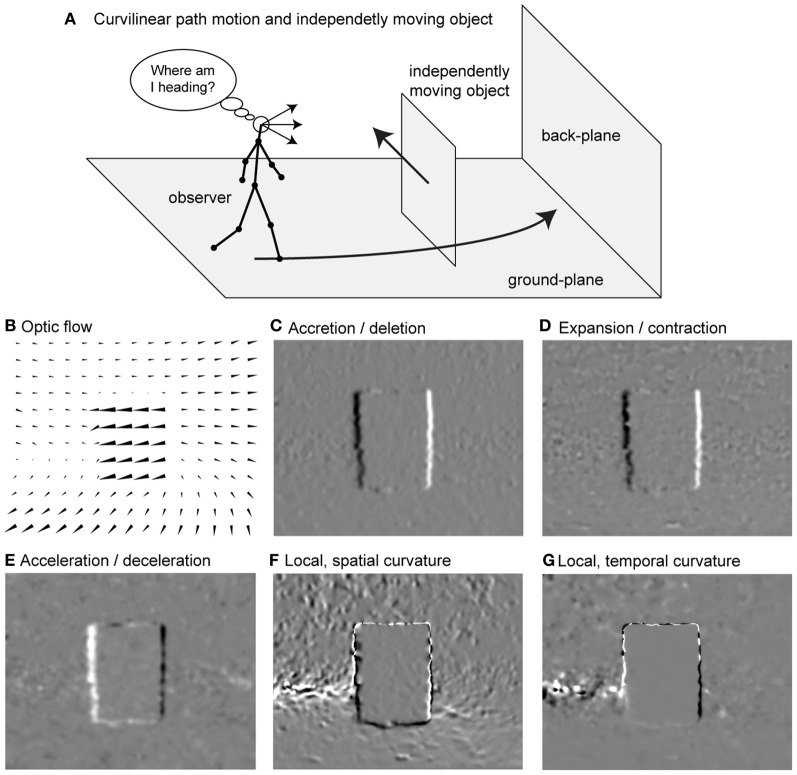
**Shows the problem of heading estimation in the presence of an IMO together with available flow-based segmentation cues. (A)** Sketch of the movements. **(B)** Detected optic flow (Zach et al., 2007). **(C)** Accretion (>0) and deletion (<0) cue. **(D)** Expansion (>0) and contraction (<0) cue. **(E)** Acceleration (>0) and deceleration (<0) cue. **(F)** Local, spatial curvature cue. **(G)** Local, temporal curvature cue. The sign indicates concave (>0) or convex (<0) curvature.

Assume an observer is on a curvilinear path while an IMO is intersecting that path, like in Figure 1A. This observer registers the optic flow as shown in Figure 1B. We propose flow-based cues for segmenting such an IMO. The first cue is accretion/deletion, see Figure 1C. This cue is present due to a faster moving object, which passes in front of a slower background. One edge of the object uncovers background, the accretion (bright), while the other edge covers or occludes background, the deletion (dark). Such cues are present at depth discontinuities and between IMOs and background. As a second cue we define expansion/contraction, see Figure 1D. Spatial derivatives in the neighborhood of these transitions in the image plane are summed. Expansion indicates a source in the flow field (bright), and contraction indicates a sink in the flow field (dark). The third cue is acceleration/deceleration, which describes the temporal characteristics of the flow at one spatial location in the image, see Figure 1E. Acceleration occurs if pixel-motion increases at the next time frame (bright) and a deceleration if pixel-motion decreases (dark). We define local, spatial curvature of flow fields as fourth cue, see Figure 1F. This cue is a local approximation of the curvature of an integral curve (a solution) interpreting the equation that describes optic flow as a system of non-linear ordinary equations (ODEs). Since this curve exists in the image plane we call it spatial, and because we use an approximation that is good only in the vicinity of the considered point, we call it local. Curvature appears either concave (bright) or convex (dark). As a fifth segmentation cue we define the local temporal curvature of the flow field, see Figure 1G. Unlike the spatial curvature, this cue describes the temporal change for a single temporally fixed location in the image. The curve is defined by the temporal change of the vector in that location.

Our goal is to derive analytical models for motion integration and segmentation when IMOs are present in the environment. We develop a linear model for the estimation of self-motion assuming linear motion and for the estimation of radius assuming curvilinear motion. Two integration mechanisms are proposed. One integrates over the entire visual field and another segments the IMO and integrates only flow from the rigid background. To establish such segmentation, we derive analytical models for the above mentioned segmentation cues (see Figures 1C–G). These cues are integrated into estimation methods of heading and path as a possible solution for the combined motion integration and segmentation.

We continue this article by describing the Methods, a description of an analytical model for optic flow, called the model of visual image motion, for different scene and motion configurations. This model is used to define and derive segmentation cues and methods estimating heading or path. Results show quantitative and qualitative evaluations of these derived models by plugging in the settings as used in the targeted psychophysical studies (Warren and Saunders, 1995; Royden and Hildreth, 1996; Fajen and Kim, 2002). The Discussion provides a classification of our analytical models in terms of existing work on heading and path perception both in modeling and in psychophysics.

## Methods

This section is organized into three parts. In the first part, we derive a motion model for linear motion toward a plane and curvilinear motion above a ground plane or toward an initially fronto-parallel plane. The second part describes methods that estimate the horizontal position of the FOE for linear motion toward a plane or the radius from curvilinear motion above a ground plane. In the third part, we define analytical models for segmentation cues of stationary and independently moving objects. Table 1 gives a summary of identifiers used in the derivations and model.

**Table 1:** **Lists all identifiers with a brief description and their physical units**.

**Identifier**	**Description**	**Unit**
*t*	Time	s
*f*	Focal length of a pinhole camera	m
(*x*, *y*, *f*)	Point on the image plane from a pinhole camera nodal point	m
*Z*(*x*, *y*, *t*)	Distance measured along the optical axis at (*x*, *y*) and t	m
$\vec{v}=\left(v_{x},\;v_{y},\;v_{z}\right)$	3D linear velocity vector	m/s
$\vec{\omega }=\left(\omega_{x},\;\omega_{y},\;\omega_{z}\right)$	3D rotational velocity vector (pitch, yaw, roll)	°/s
(*ẋ*, *ẏ*)	Model of visual image motion	m/s
β	Angle of edge normal pointing outward of the object	°
$\vec{n}(t)=\left(n_{x},\;n_{y},\;n_{z}\right)$	Normal vector of a pictured plane	None
*d*(*t*)	Distance of a pictured plane	m
*h*	Height of the camera above ground	m
ω = ω_*y*_	Rotational yaw-velocity	°/s
*v*	Linear velocity along the optical axis	m/s
*r*	Radius of curvilinear motion path	m
*t*_*c*_	Time-to-contact with a pictured plane	s
(η, ξ)	Image angle coordinates of the focus of expansion	none
Δ	Accretion (Δ > 0) and deletion (Δ < 0)	m/s
δ	Expansion (δ > 0) and contraction (δ < 0)	1/s
α	Acceleration (α > 0) and deceleration (α < 0)	m/s^2^
ϑ	Local, spatial curvature: Concave (ϑ > 0) and convex (ϑ < 0)	1/m
γ	Local, temporal curvature: Concave (γ > 0) and convex (γ < 0)	1/m
(*x*_*I*_, *y*_*I*_)	Position of the independently moving object in the image	m
(*w*_*I*_, *h*_*I*_)	Width and height of the object in the image	m
(*w*_*B*_, *h*_*B*_)	Width and height of the image plane of the pinhole camera	m

*If vectors appear in the first column, we denote the unit of each component. Some experiments indicate speeds in m/frames rather than m/s, which can be converted using the frame rate*.

In some cases we define image positions, lengths, or the x/y-components of the linear velocity in degrees of visual angle. For positions this translates into *x*_Meter_ = *f* · tan(*x*_Angle_) and for lengths *l*_Meter_ = 2 · *f* · tan(*l*_Angle_/2), where *f* denotes the focal length of a pinhole camera model. Velocities are transformed according to *v*_Meter/s_ = *v*_*z*_ · tan(*v*_Angle_), where [*v*_*z*_] = m/s is the velocity along the optical axis of the camera. Thus, we can specify positions and velocities either in degrees or degrees per second, or in meters or meters per second.

### Projection of moving 3D points onto a 2D image surface using a 3D motion model

We follow Longuet-Higgins and Prazdny's (1980) derivation of an equation for visual image motion, assuming a rigid environment, an instantaneous 3D motion, and a pinhole camera model. This derivation is briefly repeated to give an idea how our model fits and extends the model of Longuet-Higgins and Prazdny (1980).

We assume that 3D points are defined by (*X, Y, Z*) while the *z-component* is measured along the optical axis, the *x-component* to the left, and the *y*-component to the top using a right-handed coordinate system. We use uppercase letters to denote points or their components that are sampled in 3D. Three-dimensional points are projected onto the image plane of a pinhole camera. This image plane is at the distance *f* in front of the nodal point. Points projected onto this image plane are (*x*, *y*, *f*) = *f*/*Z* · (*X*, *Y*, *Z*) and we denote them by lower-case letters. We assume the 3D motion as a combination of a 3D translational velocity vector $\vec{v}={\left(v_{x},\;v_{y},\;v_{z}\right)}^{t}$ and a 3D rotational velocity vector $\vec{\omega }={\left(\omega_{x},\;\omega_{y},\;\omega_{z}\right)}^{t}$ excluding higher order temporal changes, like accelerations. We use the super-script *t* to denote the vector-transpose. According to classical kinematics these two velocities define the motion of a 3D point velocity by (*Ẋ*, *Ẏ*, *Ż*) = −(*v*_*x*_, *v*_*y*_, *v*_*z*_) − (ω_*x*_, ω_*y*_, ω_*z*_) × (*X*, *Y*, *Z*) assuming (0, 0, 0) is the center of rotation and × denotes the cross-product (Goldstein et al., 2001). Computing the temporal derivative of the projected points in the image plane and plugging in the definition of 3D point velocity yields the visual motion equation (Longuet-Higgins and Prazdny, 1980):

(1) $$\left(\begin{array}{c} \dot{x}\\ \dot{y} \end{array}\right)=\frac{1}{Z}\left(\begin{array}{ccc} -f & 0 & x\\ 0 & -f & y \end{array}\right)\left(\begin{array}{c} v_{x}\\ v_{y}\\ v_{z} \end{array}\right)+\frac{1}{f}\left(\begin{array}{ccc} xy & -(f^{2}+x^{2}) & fy\\ \left(f^{2}+y^{2}\right) & -xy & -fx \end{array}\right)\left(\begin{array}{c} \omega_{x}\\ \omega_{y}\\ \omega_{z} \end{array}\right).$$

This Equation (1) expresses the image velocity (*ẋ*, *ẏ*)^*t*^ of 3D points (*X*, *Y*, *Z*)^*t*^ that are projected onto the location (*x*, *y*)^*t*^ in the image plane. In Equation (1) we dropped the third component because its value is constantly zero.

The model of visual image motion as defined in Equation (1) has several interesting properties. First, image motion induced by linear and rotational velocity superimpose linearly. Second, only the image motion induced by linear velocity depends on the distance *Z* of the sample point. The speed of this flow is inversely proportional to depth, which means that points further away have a lower speed than points that are close. A third property is the symmetry between the *x*- and *y*-components of linear and rotational velocity and their induced image motion. For instance, when translating to the left the same image motion is generated if translating upward and flipping *x* and *y* components of the resulting image motion. The specific location (η, ζ) = (*v*_*x*_/*v*_*z*_, *v*_*y*_/*v*_*z*_) denotes the FOE. The location (ω_*x*_/ω_*z*_, ω_*y*_/ω_*z*_) is called the center of rotation, or COR (Gibson, 1950; Longuet-Higgins and Prazdny, 1980).

The computation of visual motion with Equation (1) requires the distance *Z* of surfaces from the camera expressed in the camera's coordinate frame. Thus, the distance *Z* is a function of the position in the image plane (*x*, *y*, *f*) as well as of the time *t*. It can be interpreted as the surface function *Z(x, y, t)* as seen through the pinhole camera at time *t*. The next paragraphs use Equation (1) either to further specify the surface function *Z* or to further restrict the possible paths of self-motion or object motion. This will yield the three model instances: (1) the linear motion of an observer toward a plane, (2) the curvilinear motion of an observer toward a fronto-parallel plane, and (3) the curvilinear motion of an observer above a ground-plane.

**Figure 2:**
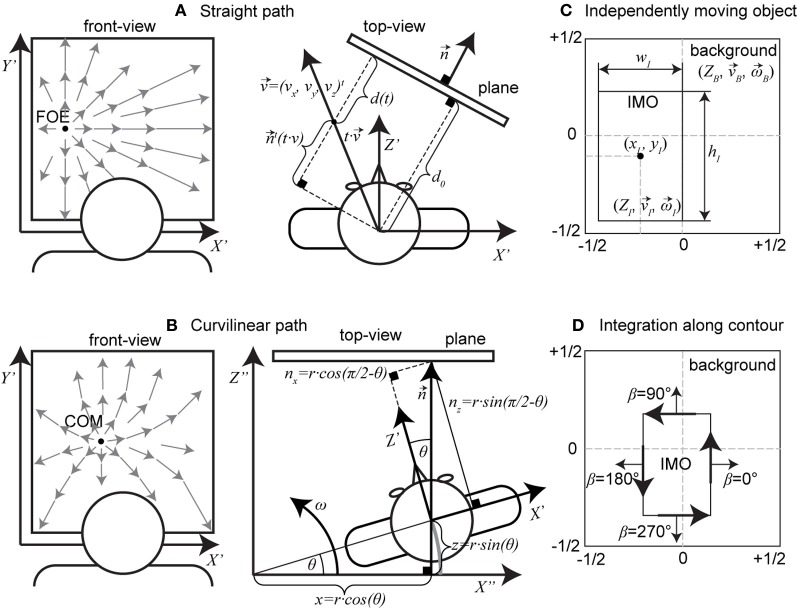
**Shows motion/scene configurations and the definitions of IMOs. (A)** A straight, linear path toward a plane; here, we show a top-down view. **(B)** Configuration for a curvilinear path toward an initially (*t* = 0 s) fronto-parallel plane, again as top-view. **(C)** Shows the definition of an IMO in the image plane by its position (*x*_*I*_, *y*_*I*_), size (*w*_*I*_, *h*_*I*_), and additional parameters $\left(Z_{I},\;{\vec{v}}_{I},\;{\vec{\omega }}_{I}\right)$. The background has the scene and motion parameters $\left(Z_{B},\;{\vec{v}}_{B},\;{\vec{\omega }}_{B}\right)$. **(D)** Integration along the contour happens using normal vectors with direction β and following the black arrows drawn on the contour.

For the first motion model, that of linear motion toward a plane, we assume that the plane is defined in Hessian normal form with the normal vector $\vec{n}=\left(n_{x},\;n_{y},\;n_{z}\right)$ and distance *d* from the origin, here the nodal point of the pinhole camera. Figure 2A shows an example of such a motion and plane configuration. We assume that the plane is stationary in 3D space. Then, the viewer-dependent distance of the plane changes with time according to

(2) $$d(t)=d_{0}-t\;\cdot \;(v_{x}\;\cdot \;n_{x}+v_{y}\;\cdot \;n_{y}+v_{z}\;\cdot \;n_{z})=d_{0}-t\;\cdot \;{\vec{v}}^{t}\vec{n}.$$

In Equation (2) *d*_0_ denotes the initial distance at *t* = 0 s. The distance *d*(*t*) changes by the distance $t\;\cdot \;\vec{v}$ traveled along the normal vector $\vec{n}$. This is formalized by projecting $t\;\cdot \;\vec{v}$ onto the normal vector $\vec{n}$ (calculating the inner vector product). The Hessian normal form for such a time variant plane reads (*n*_*x*_, *n*_*y*_, *n*_*z*_)^*t*^(*X*, *Y*, *Z*) − *d*(*t*) = 0 using an arbitrary 3D sample point (*X, Y, Z*). Plugging this equation into the projection for a pinhole camera and solving for *Z* as a function of *x*, *y*, and *t* gives:

(3) $$Z(x,\;y,\;t)=\frac{f\;\cdot \;d_{0}-f\;\cdot \;t\;\cdot \;(v_{x}\;\cdot \;n_{x}+v_{y}\;\cdot \;n_{y}+v_{z}\;\cdot \;n_{z})}{x\;\cdot \;n_{x}+y\;\cdot \;n_{y}+f\;\cdot \;n_{z}}=f\frac{d_{0}-t\;\cdot \;{\vec{v}}^{t}\vec{n}}{{\vec{p}}^{t}\vec{n}},$$

with the lower-case $\vec{p}=(x,\;y,\;f)$ being a location in the image. We use Equation (3) and plug it into Equation (1) setting all rotational velocities to zero. This gives the model instance for linear motion toward a plane:

(4) $$\left(\begin{array}{c} \dot{x}\\ \dot{y} \end{array}\right)=\frac{x\;\cdot \;n_{x}+y\;\cdot \;n_{y}+f\;\cdot \;n_{z}}{f\;\cdot \;d_{0}-f\;\cdot \;t\;\cdot \;(v_{x}\;\cdot \;n_{x}+v_{y}\;\cdot \;n_{y}+v_{z}\;\cdot \;n_{z})}\left(\begin{array}{ccc} -f & 0 & x\\ 0 & -f & y \end{array}\right)\left(\begin{array}{c} v_{x}\\ v_{y}\\ v_{z} \end{array}\right).$$

By plugging in the time-dependent surface function *Z*, we introduce a time dependency that was not explicitly stated before. In Equation (1) the only time-dependent variable is the surface function *Z*, since the focal length *f* is constant (no zoom), the sample locations (*x, y, f*) on the image plane are constant (no lens jitter or shift of the nodal point), and the linear and rotational velocities are constant (no acceleration). Studying Equation (4) further shows that it is, in general, a polynomial in *x* and *y*, and non-linear in the linear velocity components *v*_*x*_, *v*_*y*_, and *v*_*z*_.

For the second model instance, we assume curvilinear self-motion or object motion along a circular path with translational velocity $\vec{v}={\left(0,\;0,\;\omega \;\cdot \;r\right)}^{t}$ being tangent to that path and the rotational velocity $\vec{\omega }={\left(0,\;\omega ,\;0\right)}^{t}$. In this case the observer trajectory is on an orbit that is centered at an arbitrary reference in space. In addition, we assume traveling toward an initially fronto-parallel plane that appears at the distance *d*_0_. Figure 2B shows the geometry of this configuration. The normal vector of the plane changes with time and is $\vec{n}(t)={\left(-\sin(\omega \;\cdot \;t),\;0,\;\cos(\omega \;\cdot \;t)\right)}^{t}$, and so does the distance, which is *d*(*t*) = *d*_0_ − *r* · sin(ω · *t*). We use the Hessian normal form for a plane to plug in the above values combined with the perspective projection function and solve for *Z*. This result for *Z* is then plugged into Equation (1) which yields:

(5) $$\left(\begin{array}{c} \dot{x}\\ \dot{y} \end{array}\right)=\frac{x\;\cdot \;\sin(\omega \;\cdot \;t)+f\;\cdot \;\cos(\omega \;\cdot \;t)}{f\;\cdot \;(d_{0}-r\;\cdot \;\sin(\omega \;\cdot \;t))}\omega \;\cdot \;r\;\cdot \;\left(\begin{array}{c} x\\ y \end{array}\right)-\frac{\omega }{f}\left(\begin{array}{c} f^{2}+x^{2}\\ xy \end{array}\right).$$

This Equation (5) depends on the angular velocity ω and the radius *r* of the circular motion path. These two variables define the curvilinear motion. The image velocity depends on these variables in Equation (5) non-linearly.

For the third model instance we consider again curvilinear motion but change the scene geometry from a fronto-parallel plane to a ground-plane. The camera is assumed to be at the distance *d* = *h* above the ground, which has the normal vector $\vec{n}={\left(0,\;1,\;0\right)}^{t}$. In this case, the distance and normal vector do not change with time, which makes the ground-plane surface function and, thus, image velocities time-constant. Plugging these definitions into Equation (3) and plugging in the result into Equation (1) together with the definition of curvilinear motion yields:

(6) $$\left(\begin{array}{c} \dot{x}\\ \dot{y} \end{array}\right)=\frac{y}{f}\frac{r}{h}\omega \;\cdot \;\left(\begin{array}{c} x\\ y \end{array}\right)-\frac{\omega }{f}\left(\begin{array}{c} f^{2}+x^{2}\\ xy \end{array}\right).$$

Table 2 summarizes these three model instances that we use to estimate linear motion or the radius of curvilinear motion.

**Table 2:** **Lists models of visual image motion**.

**Motion**	**Scene**	**Model instance**
General	General	$\left(\begin{array}{c} \dot{x}\\ \dot{y} \end{array}\right)=\frac{1}{Z}\left(\begin{array}{ccc} -f & 0 & x\\ 0 & -f & y \end{array}\right)\left(\begin{array}{c} v_{x}\\ v_{y}\\ v_{z} \end{array}\right)+\frac{1}{f}\left(\begin{array}{ccc} xy & -(f^{2}+x^{2}) & fy\\ \left(f^{2}+y^{2}\right) & -xy & -fx \end{array}\right)\left(\begin{array}{c} \omega_{x}\\ \omega_{y}\\ \omega_{z} \end{array}\right)$
$\vec{v}=\left(v_{x},\;v_{y},\;v_{z}\right)$	*Z*(*x*, *y*, *t*)	
$\vec{\omega }=\left(\omega_{x},\;\omega_{y},\;\omega_{z}\right)$		
Translation	Plane	$\left(\begin{array}{c} \dot{x}\\ \dot{y} \end{array}\right)=\frac{x\;\cdot \;n_{x}+y\;\cdot \;n_{y}+f\;\cdot \;n_{z}}{f\;\cdot \;d_{0}-f\;\cdot \;t\;\cdot \;(v_{x}\;\cdot \;n_{x}+v_{y}\;\cdot \;n_{y}+v_{z}\;\cdot \;n_{z})}\left(\begin{array}{ccc} -f & 0 & x\\ 0 & -f & y \end{array}\right)\left(\begin{array}{c} v_{x}\\ v_{y}\\ v_{z} \end{array}\right)$
$\vec{v}=\left(v_{x},\;v_{y},\;v_{z}\right)$	$\vec{n}=\left(n_{x},\;n_{y},\;n_{z}\right)$	
$\vec{\omega }=\left(0,\;0,\;0\right)$	*d*(*t* = 0) = *d*_0_	
Curvilinear	Fronto-parallel plane	$\left(\begin{array}{c} \dot{x}\\ \dot{y} \end{array}\right)=\frac{x\;\cdot \;\sin(\omega \;\cdot \;t)+f\;\cdot \;\cos(\omega \;\cdot \;t)}{f\;\cdot \;(d_{0}-r\;\cdot \;\sin(\omega \;\cdot \;t))}\omega \;\cdot \;r\;\cdot \;\left(\begin{array}{c} x\\ y \end{array}\right)-\frac{\omega }{f}\left(\begin{array}{c} f^{2}+x^{2}\\ xy \end{array}\right)$
$\vec{v}=\left(0,\;0,\;\omega \;\cdot \;r\right)$	$\vec{n}(t=0)=\left(0,\;0,\;1\right)$	
$\vec{\omega }=\left(0,\;\omega ,\;0\right)$	*d*(*t* = 0) = *d*_0_	
Curvilinear	Ground plane	$\left(\begin{array}{c} \dot{x}\\ \dot{y} \end{array}\right)=\frac{y}{f}\frac{r}{h}\omega \;\cdot \;\left(\begin{array}{c} x\\ y \end{array}\right)-\frac{\omega }{f}\left(\begin{array}{c} f^{2}+x^{2}\\ xy \end{array}\right)$
$\vec{v}=\left(0,\;0,\;\omega \;\cdot \;r\right)$	$\vec{n}(t)=\left(0,\;1,\;0\right)$	
$\vec{\omega }=\left(0,\;\omega ,\;0\right)$	*d*(*t*) = *h*	

*We denote the motion and scene geometry as seen through a pinhole camera model. For the translation toward a plane the normal vector is independent of time; in contrast, for curvilinear motion the normal changes with time. For motion parallel to a ground-plane, normal and distance stay constant over time*.

### Estimate the horizontal FOE position for linear motion or radius for curvilinear motion

This subsection contains two derivations: the first is the derivation of a linear least squares method to estimate the horizontal FOE position (variable η), and the second is the derivation of a method to estimate the radius *r* for curvilinear motion. We use the derived estimates to evaluate their robustness in case of the presence of an IMO. This IMO is parameterized by its position, size, and 3D motion relative to a stationary camera. Figure 2C depicts these IMO parameters.

#### Estimation of horizontal FOE

We start the derivation using Equation (4) assuming that background and IMO are fronto-parallel planes, setting the normal vector to $\vec{n}={\left(0,\;0,\;1\right)}^{t}$ and putting *v*_*z*_ into the denominator on the right-hand side of the equation. We name the resulting ratios: *t*_*c*_ = *d*_0_/*v*_*z*_ the time-to-contact, η = *v*_*x*_/*v*_*z*_ the horizontal image position of the FOE, and ζ = *v*_*y*_/*v*_*z*_ the vertical image position of the FOE. We use the resulting model equation in a linear least squares approach to optimize for η and ζ:

(7) $$\begin{array}{l} F(\eta ,\;\zeta )=\iint {\left\|\left(\begin{array}{c} \dot{x}\\ \dot{y} \end{array}\right)-\gamma \left(\begin{array}{c} -f\eta +x\\ -f\zeta +y \end{array}\right)\right\|}_{2}^{2}dx\;dy\text{ with}\\ \;\gamma =\frac{1}{t_{c}-t}. \end{array}$$

This model in Equation (7) expresses the squared distance of each individual residual vector in the image plane. The residual is computed between the given optic flow (*ẋ*, *ẏ*) and a model thereof, which contains the parameters η and ζ. All residual vectors are integrated over the image plane, which is indicated by the double integral. We assume that the reciprocal γ of the temporal distance toward the wall is constant for all locations in the image plane. This assumption actually does not hold true in the presence of IMOs, which will introduce a bias. Furthermore, we assume that γ is known, e.g., estimated from the binocular disparity *c* of the background by γ(*t* = 0) = −*c*/*ċ* for *c* = *b* · *f*/*Z* with *b* being the inter-camera distance of the stereo setup. Later, we relax this assumption by instead assuming that there is a segmentation cue available that indicates the image region depicting the IMO and the image region depicting the background. To compute the solution for Equation (7) we take the partial derivatives of *F* with respect to η and ζ, set these to zero and solve for η and ζ, which gives:

(8) $$\hat{\eta }=\frac{\iint x-\dot{x}/\gamma dx\;dy}{f\;\cdot \;\iint 1\;dx\;dy\;}\text{ and }\hat{\zeta }=\frac{\iint y-\dot{y}/\gamma dx\;dy}{f\;\cdot \;\iint 1\;dx\;dy\;}$$

The solution is indicated using a hat-symbol. The solution for $\hat{\eta }$ depends on *x*, *ẋ*, *f*, and γ, and neither on *y* nor *ẏ*. Dependencies for $\hat{\zeta }$ are similar concerning the vertical axis. In the following steps we restrict the derivation to the horizontal component $\hat{\eta }$, noting that the derivation for the $\hat{\zeta }$ component is similar due to its symmetry. For the background and IMO motion we use the following model instances:

(9) $$\begin{array}{l} \left(\begin{array}{c} {\dot{x}}_{B/I}\\ {\dot{y}}_{B/I} \end{array}\right)=\mu_{B/I}\;\cdot \;\left(\begin{array}{c} -f\;\cdot \;v_{x,\;B/I}+x\;\cdot \;v_{z,\;B/I}\\ -f\;\cdot \;v_{y,\;B/I}+y\;\cdot \;v_{z,\;B/I} \end{array}\right)\text{ with}\\ \;\mu_{B/I}=\frac{1}{d_{0,\;B/I}-t\;\cdot \;v_{z,\;B/I}}. \end{array}$$

Equation (9) defines the visual motion for the background assuming self-motion and for the IMO composed of self-motion and object motion. The subscript *B* indicates the visual motion and parameters for the background and the subscript *I* the visual motion and parameters of the IMO.

To define the registered analytical optic flow we use the two model instances from Equation (9) that define the flow for the IMO region and background in the image plane. The linear method estimating the horizontal FOE position, see Equation (8), integrates all flows within the image plane. Per definition of the analytical flow, we split this integration into two areas one for the IMO and one for the remaining background. These areas for IMO and background are defined by:

(10a) $$A_{I}=\left[x_{I}-w_{I}/2,\;x_{I}+w_{I}/2\right]\times \left[y_{I}-h_{I}/2,\;y_{I}+h_{I}/2\right]$$

(10b) $$A_{B}=\left[-w_{B}/2,\;w_{B}/2\right]\times \left[-h_{B}/2,\;h_{B}/2\right]\backslash A_{\text{I}}.$$

The size of the image plane is *w*_*B*_ × *h*_*B*_ and the size of the IMO is *w*_*I*_ × *h*_*I*_. We weigh the IMO area by the segmentation signal (1-*s)* to discount the IMO's flow within integration. To simplify the derivation, we use the auxiliaries *I*_1_ and *I*_2_ for background and IMO motion as well as their respective areas:

(11) $$I_{1}=\iint x-\dot{x}/\gamma \;dx\;dy\text{ and }I_{2}=f\;\cdot \;\iint 1\;dx\;dy.$$

The dependency on background or IMO motion always coincides with the respective motions and, thus, is implicitly contained in the 2nd sub-index. For instance, I_1, *B*_ is the evaluation of the 1st integral expression integrating background motion within the background area, and I_1, *I*_ is the evaluation of the 1st integral expression integrating IMO motion within the IMO area. Splitting the integration according to the different motions and summing the evaluated integrals gives:

(12) $$\hat{\eta }=\frac{I_{1,\;B,\;AB}+(1-s)\;\cdot \;I_{1,\;I,\;AI}}{I_{2,\;B,\;AB}+(1-s)\;\cdot \;I_{2,\;I,\;AI}}.$$

The integrals in Equation (12) can be evaluated analytically since all functions are polynomials in *x* and *y*. We define the heading bias in horizontal, linear motion as estimate minus the background motion: $\Delta \eta =\hat{\eta }-\eta_{B}$. Plugging in all evaluated integrals, we get:

(13) $$\Delta \eta =\hat{\eta }-\eta_{B}=(1-s)\frac{w_{I}h_{I}}{w_{B}h_{B}}\times \left(-\frac{v_{x,\;B}}{v_{z,\;B}}+\frac{x_{I}}{f}-\frac{\mu_{I}}{\mu_{B}}\frac{v_{x,\;B}}{v_{x,\;I}}+\frac{x_{I}}{f}\frac{\mu_{I}}{\mu_{B}}\frac{v_{z,\;I}}{v_{z,\;B}}\right).$$

Intuitively, the bias is introduced because the registered, analytical flow contains an IMO, while the model from Equation (8) assumes self-motion in a rigid environment. Assuming a full segmentation (*s* = 1), the bias vanishes. The bias term has several properties: it is proportional to the area of the IMO while keeping other parameters constant; and it depends on the horizontal position *x*_*I*_ of the IMO, the focal length *f*, the ratio of reciprocal distance values μ_*I*_ and μ_*B*_, and also the motion components of IMO and background. This heading bias is without units because η is defined as the ratio of two velocity components. Furthermore, the bias Δη is independent of the vertical position of the IMO and the vertical motion components of background *v*_*y*, *B*_ and IMO *v*_*y*, *I*_.

#### Estimation of radius for curvilinear motion

Our goal is to find a constraint for a linear estimation of the radius given the model instance of ground-plane motion [see Equation (6)] and to use this linear model to derive a bias term when there is an IMO. In the first part we derive a linear model and in the second part a bias term based on that model. The bias term involves the definition of analytical flow for the IMO, a fronto-parallel plane, and background. By dividing Equation (6) through the rotational velocity ω, we get:

(14) $$\frac{1}{\omega }\;\cdot \;\left(\begin{array}{c} \dot{x}\\ \dot{y} \end{array}\right)=\frac{y}{f}\frac{r}{h}\;\cdot \;\left(\begin{array}{c} x\\ y \end{array}\right)-\frac{1}{f}\left(\begin{array}{c} f^{2}+x^{2}\\ xy \end{array}\right).$$

To estimate *r*, we multiply this Equation (14) by the vector (−*ẏ*, *ẋ*) which cancels the dependency on the rotational velocity ω. Solving Equation (14) for *r* yields the linear constraint we were looking for:

(15) $$\hat{r}=h\;\cdot \;\frac{\dot{x}\;\cdot \;xy-(f^{2}+x^{2})\;\cdot \;\dot{y}}{\dot{x}\;\cdot \;y^{2}-xy\;\cdot \;\dot{y}}.$$

With this estimate of *r* the path can be predicted in the image plane assuming that heading is tangent to the trajectory. The predicted path is given by the curve:

(16) $$\left(\begin{array}{c} x_{c}(t)\\ y_{c}(t) \end{array}\right)=f\left(\begin{array}{c} 1/\text{​}\sin(\omega t)-\tan(\omega t)\\ -h/r\;\cdot \;1/\left|\sin(\omega t)\right| \end{array}\right).$$

The absolute value in the definition of the *y*-component accounts for negative angular velocity. In the limit ω · *t* → π/2 the *y*-component reaches the value of −*h*/*r*. This is the point of the circular path furthest away from its center around which the observer is orbiting.

To improve the estimate of *r*, we separately integrate the numerator and denominator of Equation (16) assuming that these are independent measurements. Thus, we define the two integral expressions:

(17) $$\begin{array}{l} I_{3}=\iint \dot{x}\;\cdot \;xy-(f^{2}+x^{2})\;\cdot \;\dot{y}\;dx\;dy\text{ and}\\ I_{4}=\iint \dot{x}\;\cdot \;y^{2}-xy\;\cdot \;\dot{y}\;dx\;dy. \end{array}$$

For instances of the motion model we plug in Equation (5) for the IMO motion and Equation (6) for the background motion, adding the sub-script *B* and *I*, respectively, to the path parameters ω and *r*. We sum the evaluated integrals from Equation (17) as before in Equation (12). We define the bias for curvilinear path motion ρ_*r*_ based on the ratio between the estimated radius $\hat{r}$ to the radius of the background motion *r*_*B*_. An evaluation of all terms yields the bias:

(18) $$\rho_{r}=\frac{\hat{r}}{r_{B}}=\frac{\frac{\omega_{B}}{\omega_{I}}\left(w_{B}h_{B}^{3}-w_{I}{\left(2y_{I}-h_{I}\right)}^{3}\right)+24\;\cdot \;(1-s)f\frac{h}{d_{0}}\frac{r_{I}}{r_{B}}w_{I}h_{I}y_{I}}{\frac{\omega_{B}}{\omega_{I}}\left(w_{B}h_{B}^{3}-w_{I}{\left(2y_{I}-h_{I}\right)}^{3}\right)+2\;\cdot \;(1-s)\;\cdot \;w_{I}h_{I}\left(12y_{I}^{2}+h_{I}^{2}\right).}$$

Intuitively, this bias term is introduced by assuming all flow to be generated by self-motion above a ground plane, see Equations (15) and (17). However, the IMO is neither rigidly connected to the background nor does it move with the same parameters as the background. Thus, an analytical evaluation of the integrals according to the registered flow for IMO and background area separately yields a deviation from the expected self-motion model. Properties of this bias are: It has no units; it is independent of the horizontal position *x*_*I*_ of the IMO; and for a full segmentation it evaluates to the value one. Table 3 summarizes the two bias terms from Equation (13) to (18). Thus far, we have integrated motion over the entire image plane. Next, we introduce motion segmentation cues, deriving analytical models for such cues. These cues can encode information about motion parallax, independent motion, and background motion.

**Table 3:** **Lists the bias terms for translational motion toward fronto-parallel planes and curvilinear motion above a ground plane where an IMO is modeled as fronto-parallel plane**.

**Scenario**	**Bias term**
Translational toward fronto-parallel planes	$\Delta \eta =(1-s)\frac{w_{I}h_{I}}{w_{B}h_{B}}\left(-\frac{v_{x,\;B}}{v_{z,\;B}}+\frac{x_{I}}{f}-\frac{\mu_{I}}{\mu_{B}}\frac{v_{x,\;B}}{v_{x,\;I}}+\frac{x_{I}}{f}\frac{\mu_{I}}{\mu_{B}}\frac{v_{z,\;I}}{v_{z,\;B}}\right)\text{ with }\mu_{I}=\frac{1}{d_{0,\;I}-t\;\cdot \;v_{z,\;I}}\text{ and }\mu_{B}=\frac{1}{d_{0,\;B}-t\;\cdot \;v_{z,\;B}}$
Curvilinear path motion above a ground plane and IMO as fronto-parallel plane	$\rho_{r}=\frac{\frac{\omega_{B}}{\omega_{I}}\left(w_{B}h_{B}^{3}-w_{I}{\left(2y_{I}-h_{I}\right)}^{3}\right)+24\;\cdot \;(1-s)f\frac{h}{d_{0}}\frac{r_{I}}{r_{B}}w_{I}h_{I}y_{I}}{\frac{\omega_{B}}{\omega_{I}}\left(w_{B}h_{B}^{3}-w_{I}{\left(2y_{I}-h_{I}\right)}^{3}\right)+2\;\cdot \;(1-s)\;\cdot \;w_{I}h_{I}\left(12y_{I}^{2}+h_{I}^{2}\right)}$

### Analytical models for segmentation cues in visual motion

In this subsection the goal is to derive analytical models of segmentation cues in order to integrate only over the background, rather than the entire visual image plane that may contain regions with IMOs. Furthermore, segmentation cues can help to develop a qualitative understanding of the scene in terms of rigidity or depth order. Inspired by psychophysics (Gibson, 1950; Koenderink and van Doorn, 1976; van Doorn and Koenderink, 1982; Braddick, 1993), neurophysiology (Orban et al., 1992; Eifuku and Wurtz, 1998; Orban, 2008), imaging studies (Zeki et al., 2003; Bartels et al., 2008), and modeling work (Barnes and Mingolla, 2012), we define five distinct segmentation cues.

The first cue is the accretion and deletion cue. We incorporate into our definition of accretion/deletion the orientation of the local edge formed between object and background since locally we only notice the amount of accretion/deletion that happens normal to that edge. We define the normal of the edge to point outward of the IMO and this normal is defined by the angle β. To encapsulate all these described properties, we define accretion/deletion by:

(19) $$\Delta =\Delta_{B}-\Delta_{I}\text{ with }\Delta_{B/I}=\left({\dot{x}}_{B/I},\;{\dot{y}}_{B/I}\right)\left(\begin{array}{c} \cos(\beta )\\ \sin(\beta ) \end{array}\right).$$

Equation (19) can be used by any instance of the motion model in Table 2 by plugging in the respective motion parameters for background and IMO. If the IMO's motion to the right is faster than the background to the right, then a deletion occurs (Δ < 0) at the right, vertical edge of the IMO. On left vertical edge of the IMO an accretion occurs (Δ > 0). The unit of accretion and deletion is m/s.

As a second cue we utilize the local expansion/contraction of the flow field. This cue is motivated by the divergence definition for vector fields. Consider the Jacobian matrix **J** computed from the vector field (*ẋ*(*x*, *y*, *t*), *ẏ*(*x*, *y*, *t*))^*t*^ by taking the partial derivatives of *ẋ* and *ẏ* with respect to *x* and *y*. The expansion/contraction cue can then be determined from the trace of the Jacobian, trace(**J**), namely

(20) $$\delta =\delta_{I}-\delta_{B}\text{ with }\delta_{B/I}=\partial_{x}{\dot{x}}_{B/I}+\partial_{y}{\dot{y}}_{B/I}.$$

We assume that these derivatives are locally computed in the neighborhoods of an image location (*x, y*) that falls onto the edge between IMO and background. Then, the difference between the locally evaluated cue for the IMO and for the background is computed. If the vectors that belong to the IMO or object, in general, form a radially outward pointing pattern like a source in a vector field, and if such pattern is faster than that for the background, the overall cue indicates an expansion (δ > 0). An expansion pattern exists also, if the background has the characteristic of a sink point while the IMO or object appears stationary or slower than the background. Otherwise, the overall cue is given as sink point (δ < 0). The unit for this cue is 1/s or Hz. This expansion/contraction cue has been studied and discussed in the psychophysical literature (Koenderink and van Doorn, 1976; Braddick, 1993).

We define accelerations/decelerations as a third cue. These are 2nd order temporal derivatives of points projected onto the image plane much like the acceleration and deceleration known from classical mechanics (Goldstein et al., 2001). Considering such derivatives are motivated by studies demonstrating sensitivity to such cues (Angelaki et al., 1993; Cao et al., 2004). Similarly to the accretion and deletion cue, we define acceleration and deceleration along the normal of a local edge that is defined by the angle β. A combination of these desired properties leads to the definition:

(21) $$\alpha =\alpha_{I}-\alpha_{B}\text{ with }\alpha_{B/I}=\left({\ddot{x}}_{B/I},\;{\ddot{y}}_{B/I}\right)\left(\begin{array}{c} \cos(\beta )\\ \sin(\beta ) \end{array}\right).$$

This cue indicates acceleration for α > 0 and it indicates deceleration for α < 0. The units of this cue are m/s^2^.

The fourth cue is defined as local, spatial curvature. We stress the fact that this is a local cue, since we use a Taylor series approximation up to the 2nd order to derive it. Thus, the local curve in the image plane is defined by:

(22) $$\lambda (t)=\left(\begin{array}{c} x\\ y \end{array}\right)+\left(\begin{array}{c} \dot{x}\\ \dot{y} \end{array}\right)\;\cdot \;t+\frac{1}{2}\left(\begin{array}{cc} \partial_{x}\dot{x} & \partial_{y}\dot{x}\\ \partial_{x}\dot{y} & \partial_{y}\dot{y} \end{array}\right)\left(\begin{array}{c} \dot{x}\\ \dot{y} \end{array}\right)\;\cdot \;t^{2}.$$

We use this local approximation since the analytical solution [*x*(*t*), *y*(*t*)] for the non-linear, coupled system of two ordinary differential equations (ODEs) as defined by Equation (1) might be unknown. Assuming our local approximation, the curvature is computed by $\det(\dot{\lambda },\;\ddot{\lambda })/{\left\|\dot{\lambda }\right\|}^{3}$. Plugging in the definition of Equation (22) yields:

(23) $$\begin{array}{l} \;\vartheta =\vartheta_{I}-\vartheta_{B}\text{ with}\\ \vartheta_{B/I}=\frac{\left|\begin{array}{cc} {\dot{x}}_{B/I} & {\dot{x}}_{B/I}\;\cdot \;(\partial_{x}{\dot{x}}_{B/I})+{\dot{y}}_{B/I}\;\cdot \;(\partial_{y}{\dot{x}}_{B/I})\\ {\dot{y}}_{B/I} & {\dot{x}}_{B/I}\;\cdot \;(\partial_{x}{\dot{y}}_{B/I})+{\dot{y}}_{B/I}\;\cdot \;(\partial_{y}{\dot{y}}_{B/I}) \end{array}\right|}{{\sqrt{{\dot{x}}_{B/I}^{2}+{\dot{y}}_{B/I}^{2}}}^{3}}. \end{array}$$

The Equation (23) defines the curvature difference between IMO and background. The units of this curvature are 1/m. This curvature can be interpreted as the curvature of integral curves that are solutions to the system of ODEs in Equation (1). This curve has a concave shape for ϑ > 0 and a convex shape for ϑ < 0.

As a fifth cue for segmentation we define local, temporal curvature. In contrast to the local, spatial curvature that is defined in the image plane, this curvature is defined for a single location in the visual field along the temporal domain. This assumes that the curve is defined as (*ẋ*(*t*), *ẏ*(*t*)) for one fixed image location. Thus, the curvature is:

(24) $$\gamma =\gamma_{I}-\gamma_{B}\text{ with }\gamma_{B/I}=\frac{{\dot{x}}_{B/I}\;\cdot \;{\ddot{y}}_{B/I}-{\dot{y}}_{B/I}\;\cdot \;{\ddot{x}}_{B/I}}{{\sqrt{{\dot{x}}_{B/I}^{2}+{\dot{y}}_{B/I}^{2}}}^{3}}.$$

This curve has a concave shape for γ > 0 and a convex shape for γ < 0. The units for this curvature are 1/m. Table 4 summarizes the definition for these five segmentation cues.

**Table 4:** **Defines analytical models for five segmentation cues of independently moving objects or stationary objects in the scene**.

**Cue**	**Model term**
Accretion (Δ > 0) and deletion (Δ < 0)	$\Delta =\Delta_{B}-\Delta_{I}\text{ with }\Delta_{B/I}={\left(\begin{array}{c} {\dot{x}}_{B/I}\\ {\dot{y}}_{B/I} \end{array}\right)}^{t}\left(\begin{array}{c} \cos(\beta )\\ \sin(\beta ) \end{array}\right)$
Expansion (δ > 0) and contraction (δ < 0)	$\delta =\delta_{I}-\delta_{B}\text{ with }\delta_{B/I}=\partial_{x}{\dot{x}}_{B/I}+\partial_{y}{\dot{y}}_{B/I}$
Acceleration (α > 0) and deceleration (α < 0)	$\alpha =\alpha_{I}-\alpha_{B}\text{ with }\alpha_{B/I}={\left(\begin{array}{c} {\ddot{x}}_{B/I}\\ {\ddot{y}}_{B/I} \end{array}\right)}^{t}\left(\begin{array}{c} \cos(\beta )\\ \sin(\beta ) \end{array}\right)$
Local, spatial curvature: concave (ϑ > 0) and convex (ϑ < 0)	$\vartheta =\vartheta_{I}-\vartheta_{B}\text{ with }\vartheta_{B/I}=\frac{\left|\begin{array}{cc} {\dot{x}}_{B/I} & {\dot{x}}_{B/I}\;\cdot \;(\partial_{x}{\dot{x}}_{B/I})+{\dot{y}}_{B/I}\;\cdot \;(\partial_{y}{\dot{x}}_{B/I})\\ {\dot{y}}_{B/I} & {\dot{x}}_{B/I}\;\cdot \;(\partial_{x}{\dot{y}}_{B/I})+{\dot{y}}_{B/I}\;\cdot \;(\partial_{y}{\dot{y}}_{B/I}) \end{array}\right|}{{\sqrt{{\dot{x}}_{B/I}^{2}+{\dot{y}}_{B/I}^{2}}}^{3}}$
Local, temporal curvature: concave (γ > 0) and convex (γ < 0)	$\gamma =\gamma_{I}-\gamma_{B}\text{ with }\gamma_{B/I}=\frac{{\dot{x}}_{B/I}\;\cdot \;{\ddot{y}}_{B/I}-{\dot{y}}_{B/I}\;\cdot \;{\ddot{x}}_{B/I}}{{\sqrt{{\dot{x}}_{B/I}^{2}+{\dot{y}}_{B/I}^{2}}}^{3}}$

*The sign of these defined expressions has the interpretation as indicated in the left column. The local, spatial curvature is approximated by a Taylor series expansion up to the 2nd order*.

We evaluate the segmentation cues for the scene and motion configurations as given in Warren and Saunders (1995), Royden and Hildreth (1996), and Fajen and Kim (2002) and summarize the analytical expressions for all cases and experimental configurations in Table 5. In the case of linear motion curvature cues (ϑ or γ) are absent, and in the case of curvilinear motion above a ground-plane cues of acceleration/deceleration and local, temporal curvature (α and γ) are absent; Table 5 shows that the cue values are set to zero in these cases.

**Table 5:** **Provides the analytical models for the five segmentation cues and three experimental conditions**.

**Motion/scene model**	**Translation/fronto-parallel plane**	**Curvilinear/fronto-parallel plane**	**Curvilinear/ground plane**
Accretion/deletion	$\Delta =\frac{1}{d_{0}}((-f\;\cdot \;v_{x}+x\;\cdot \;v_{z})\cos(\beta )+\;(-f\;\cdot \;v_{y}+y\;\cdot \;v_{z})\sin(\beta ))$	$\Delta =\frac{\omega \;\cdot \;r}{d_{0}}\left(x\;\cdot \;\cos(\beta )+y\;\cdot \;\sin(\beta )\right)-\frac{\omega }{f}\left(\left(f^{2}+x^{2})\;\cdot \;\cos(\beta )+xy\;\cdot \;\sin(\beta \right)\right)$	$\Delta =\frac{y}{f}\frac{r}{h}\omega \;\cdot \;\left(x\;\cdot \;\cos(\beta )+y\;\cdot \;\sin(\beta )\right)-\frac{\omega }{f}\left(\left(f^{2}+x^{2})\;\cdot \;\cos(\beta )+xy\;\cdot \;\sin(\beta \right)\right)$
Expansion/contraction	$\delta =\frac{2\;\cdot \;v_{z}}{d_{0}}$	$\delta =\omega \;\cdot \;\left(\frac{2r}{d_{0}}-\frac{3x}{f}\right)$	$\delta =3\omega \;\cdot \;\left(\frac{y}{f}\frac{r}{h}-\frac{x}{f}\right)$
Acceleration/deceleration	$\alpha =\frac{v_{z}}{d_{0}^{2}}((-f\;\cdot \;v_{x}+x\;\cdot \;v_{z})\cos(\beta ))+\;(-f\;\cdot \;v_{y}+x\;\cdot \;v_{z})\sin(\beta ))$	$\alpha =\frac{x\;\cdot \;d_{0}+r\;\cdot \;f}{f\;\cdot \;d_{0}^{2}}\omega^{2}r\;\cdot \;(x\cos(\beta )\;+y\sin(\beta ))$	α = 0
Local, spatial curvature	ϑ = 0	$\vartheta =\frac{\frac{y}{f\;\cdot \;d_{0}^{2}}\left(fxr-d_{0}(f^{2}+x^{2})\right)}{\sqrt{{\left(\frac{r\;\cdot \;x}{d_{0}}-\frac{f^{2}+x^{2}}{f}\right)}^{2}+{\left(\frac{r\;\cdot \;y}{d_{0}}-\frac{x\;\cdot \;y}{f}\right)}^{2}}}$	$\vartheta =\frac{\frac{y}{f\;\cdot \;h^{2}}(y^{2}r^{2}-2hxyr+h^{2}x^{2}+f^{2}h^{2})}{\sqrt{{\left(\frac{y}{f}\frac{r\;\cdot \;x}{h}-\frac{f^{2}+x^{2}}{f}\right)}^{2}+{\left(\frac{y}{f}\frac{r\;\cdot \;y}{h}-\frac{x\;\cdot \;y}{f}\right)}^{2}}}$
Local, temporal curvature	γ = 0	$\gamma =\frac{\frac{y}{d_{0}^{2}}r\;\cdot \;(f\;\cdot \;r-x\;\cdot \;d_{0})}{\sqrt{{\left(\frac{r\;\cdot \;x}{d_{0}}-\frac{f^{2}+x^{2}}{f}\right)}^{2}+{\left(\frac{r\;\cdot \;y}{d_{0}}-\frac{x\;\cdot \;y}{f}\right)}^{2}}}$	γ = 0

*Some cues are absent for certain motion/scene settings, in which case the cue values are set to zero*.

We further evaluate these segmentation cues from Table 5 for their contribution to an overall segmentation of an IMO from the background. Figure 2D shows the integration along the contour of an IMO while considering the normal vector of the local edge. A combination of values of each of the four line segments is achieved by summing their absolute value. We take the absolute value for the following reason: assume an object has a leftward motion and appears in front of a rightward moving background. This gives an accretion on the right edge of the IMO and deletion on the left edge of the IMO. The sum results in a zero-net segmentation. To avoid such a zero-sum result, we take the absolute value for contributions of each line segment. Due to taking the absolute value, the interpretation of the sign that we provided in Table 5 is no longer valid since all values are positive. We evaluate the segmentation cue along the contour using the following integrals:

(25) $$\begin{array}{l} s_{\nu }=\left|\int_{y_{I}-h_{I}/2}^{y_{I}+h_{I}/2}f_{\nu }(x_{I}+w_{I}/2,\;y;\;\beta =0^{{}^{\circ}})\;dy\right|\\ \;+\left|\int_{x_{I}+w_{I}/2}^{x_{I}-w_{I}/2}f_{\nu }(x,\;y_{I}+h_{I}/2;\;\beta =90^{{}^{\circ}})\;dx\right|\\ \;+\left|\int_{y_{I}+h_{I}/2}^{y_{I}-h_{I}/2}f_{\nu }(x_{I}-w_{I}/2,\;y;\;\beta =180^{{}^{\circ}})\;dy\right|\\ \;+\left|\int_{x_{I}-w_{I}/2}^{x_{I}+w_{I}/2}f_{\nu }(x,\;y_{I}-h_{I}/2;\;\beta =270^{{}^{\circ}})\;dx\right|. \end{array}$$

In Equation (25) we use the generic function *f*_ν_ that can be any of the cues ν ε {Δ, δ, α, ϑ, γ} defined in Table 5. Note that this function depends on the image location (*x*, *y*) and in cases of vector-valued cues also on the orientation of the edge normal β. For the definition of the combined segmentation cue, we use a linear superposition of the segmentation cues accretion/deletion, expansion/contraction, and acceleration/deceleration. Thus, the combined segmentation cue is:

(26) $$s=c_{\Delta }\;\cdot \;s_{\Delta }+c_{\delta }\;\cdot \;s_{\delta }+c_{\alpha }\;\cdot \;s_{\alpha }$$

with coefficients *c*_Δ_, *c*_δ_, and *c*_α_ that are altered to fit the data. We incorporate this segmentation cue into methods estimating the horizontal FOE position or path radius by weighting integrals ranging over background and IMO regions in the image plane accordingly. IMO regions are weighted by (1-*s*) where *s* ranges between zero and one. For a full segmentation, *s* = 1, this discounts the region where the IMO is present for the estimation of the background motion. The Equation (26) does not include available curvature cues, since analytical expressions for the corresponding line integrals are difficult to find.

## Results

We organized the results into four subsections, beginning with a description of heading bias and path perception for estimation methods which do not use segmentation.

### Linear least square estimation of heading or path (radius) cannot explain observed behavior

We study the validity of analytically derived bias terms for translational and curvilinear path motion by comparison to related psychophysical studies (Warren and Saunders, 1995; Royden and Hildreth, 1996; Fajen and Kim, 2002).

In the first set of experiments, following Warren and Saunders (1995), the scene is defined by two fronto-parallel planes, one for the background and one for the IMO, both at an initial (*t* = 0) distance of *d*_*B*, 0_ = *d*_*I*, 0_ = 10 m. Their speeds along the optical axis are *v*_*z*, *B*_ = 2 m/s and *v*_*z*, *I*_ = 3 m/s, respectively. The heading direction of the background appears in the horizontal at any combination of probe locations at ±10°, ±8°, ±6°, or ±4° and a variable heading around that probe of ±4°, ±2°, ±1°, or ±0.5°. This gives 16 combinations at which background heading can appear. In addition, the IMO appears at a horizontal angle ±6° at the opposite side of the probe. The path angle is defined as the angular difference between the motion direction of background and the motion direction of the IMO. These experiments probe horizontal path angles of −6°, 0°, and +6°. All these parameters assume that motions are defined with respect to a stationary camera. Our model assumes an instantaneous flow field (snapshot) as it is present at the end of each stimulus presentation. For simplicity, we excluded a temporal integration in our analytical models. The stimulus is present for 1.5 s. Thus, the size of the IMO at the end of the stimulus presentation has increased by a factor of 1.6 due to looming. At the end of each stimulus presentation the IMO has a size of 16° × 16°. For the model we use the display size of 32° × 32° or *w*_*B*_ = 1 cm and *h*_*B*_ = 1 cm with a focal length of *f* = 1.74 cm. The original study used a display size of 40° × 32°. We simplified the model by using a square display.

**Figure 3:**
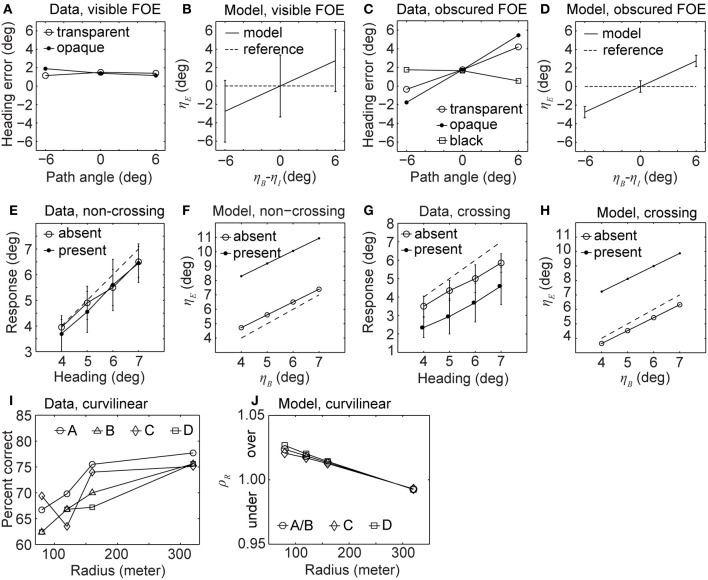
**Displays heading biases and perceived path from experimental data (Warren and Saunders, 1995; Royden and Hildreth, 1996; Fajen and Kim, 2002) and our analytical model without segmentation cues cannot explain the data completely. (A,B)** Show the heading error for humans and our model, respectively, when the FOE is visible. **(C,D)** Show the heading error for humans and our model, respectively, when the FOE is covered by the IMO. **(E,F)** Show the estimated heading for humans and our model, respectively, when the IMO's path and the observer's path are not crossing. **(G,H)** Show the estimated heading for humans and our model, respectively, for crossing paths. **(I)** Percent correct responses of humans estimating future path for four different configurations labeled as A, B, C, and D. **(J)** Error of the radius estimated by our model for the same configurations.

Figures 3A–D shows the data and model results side by side. All four graphs report the heading error over path angle. The heading error in the model is denoted by η_*E*_ and the path angle by η_*B*_−η_*I*_. The reporting of heading bias is divided into two groups. In one group the FOE of the background is visible and in the other group, the FOE of the background is obscured by the IMO. In the visible case, the heading error for humans is small with a constant bias underestimating the heading. This means that the FOE of the background is estimated to be closer to the center of the screen, see Figure 3A. Open circles in the graph of Figure 3A depict the error for an opaque IMO, graphs with closed circles encode the error for a transparent IMO. This underestimation of heading is a known effect reported in studies without IMOs (Warren and Kurtz, 1992; Crowell and Banks, 1993). In the model, heading errors appear large although cases of zero error appear within the standard deviation, regardless of the path angle, see Figure 3B. For the case of the obscured FOE humans have a bias in heading perception, see Figure 3C. In the black condition (squared symbols) the IMO region is blanked out such that no texture appears in this region. The model replicates this behavior except for the shift in estimating heading closer to the center of the screen, which is not an effect of the IMO, see Figure 3D.

The second set of experiments follows the study of Royden and Hildreth (1996). In their experimental design background and IMO appear as fronto-parallel planes. The background is initially at the distance *d*_0, *B*_ = 10 m and the IMO stays constantly at the distance *d*_0, *I*_ = 5 m, moving along with the observer. However, the IMO changes its horizontal distance from the observer by moving sideways with a velocity of 8.1°/s (0.14 rad/s) in the image plane. For the frame rate of 25 Hz, as used in the experiment, this translates into a horizontal velocity of *v*_*x*, *I*_ = 5 m · tan(0.14 rad/s/25 Hz) · 25 Hz or *v*_*x*, *I*_ = 0.71 m/s. Assuming a stationary observer, the background moves by *v*_*z*, *B*_ = 2 m/s. In the experiment the FOE for the background appears at −2°, 0°, or +2° vertically and 4°, 5°, 6°, or 7° horizontally. The field of view is 30° × 30°. In our model we choose a display size of 1 cm × 1 cm and adjusted the focal length for a 30° visual field, *f* = 1.87 cm. The experiment is composed of two conditions. In the non-crossing condition the IMO's path does not cross the observer's path and in the crossing condition it does. For the non-crossing condition the IMO starts at the horizontal position *x*_*I*_ = 0.6° and for the crossing condition it starts at *x*_*I*_ = 10.7°. In all trials, the projected size of the IMO stays constant with 10° × 10°.

Figures 3E–H shows the reported heading of the observers in the presence of an IMO from psychophysical experiments and our analytical model. In the non-crossing condition heading errors for humans are small; see Figure 3E. Our analytical model instead shows large heading errors; see Figure 3F. Note the difference in axis scaling between Figures 3E,F. In the crossing condition the perceptually reported heading error is large and tends to underestimate the true heading, dashed line in Figure 3G. Again the model's error is larger, see Figure 3H.

The third set of experiments studies path perception, following the study of Fajen and Kim (2002). The observer and IMO orbit. From the viewpoint of the observer or IMO, this is a curvilinear motion. In all conditions the background (or observer) moves with the constant, linear velocity *v*_*B*_ = 13.2 m/s, and the rotational velocity changes according to the radii *r*_*B*_ = 80 m, 120 m, 160 m, and 320 m. For these changing radii the rotational velocity is ω = *v*/*r*; or ω_*B*_ = 9.5°/s, 6.3°/s, 4.7°/s, and 2.4°/s. The rotational velocity of the IMO is 2.86°/s slower than that of the background. Thus, the object does not approach the observer as fast as the background does. It moves from near to far while the background moves from far to near. We test four different IMO paths: the IMO starts and remains to the right (path A) or to the left (path B) of the observer, or the IMO's radius decreases after initially appearing to the right (path C) or to the left (path D) of the observer. In the last two conditions, the IMO's path and the observer's paths are crossing, and 2 s after the start, the radius of the IMO either increased or decreased by 7.2 m. Thus, *r*_*I*_ = *r*_*B*_ ± 7.2 m. For the evaluation of the analytical model we assume an eye height of *h* = −1.65 m and a gaze parallel to the ground. Furthermore, we assume the IMO appears at *y*_*I*_ = −5° below the horizon and is represented as a fronto-parallel plane 5 m away from the observer, covering 5° × 5° of the observer's visual field. Fajen and Kim (2002) did not describe these IMO parameters for their study. We assume a square display of 32° × 32° to simplify the analytical model instead of 42° × 32° used in the experiment. The procedure of the experiment included humans indicating their future path by reporting whether they would pass a presented pole left or right. The accuracy of path perception was reported as a percentage of correct responses percentage over all trials.

Figures 3I,J show results of curvilinear path perception for humans and our analytical model. Between conditions (path A–D), the correct detection rate is not significantly different, see Figure 3I. However, trajectories with high curvature (small *r*) are more difficult to predict than those of low curvature (large *r*). For the analytical model we report the ratio ρ_*r*_ of estimated radius *r*_*E*_ to ground-truth radius *r*_*B*_ of the background motion: ρ_*r*_ = *r*_*E*_/*r*_*B*_. We note that the future path can be predicted solely based on the radius of the circular motion, assuming that this radius stays constant with time and that the optical axis is tangent to the trajectory; see Equation (16) in the Methods. Therefore, we assume the error measure is informative about the paths chosen by humans. We depict the error for our analytical model in Figure 3J. Errors above one indicate an overestimation, errors below one an underestimation, and values close to one represent a small error. Paths A and B have the same error and only one curve is plotted. Note that the trend for the model is the same as in the data, compare Figures 3I,J. High curvatures result in large errors or a low correct detection rate. Low curvatures have small errors or a high correct detection rate.

Thus far, our analytical model does not correctly explain all psychophysical data. One shortcoming of the model is the integration of motion across the entire image including IMOs; however, IMOs can be segmented and, thus, effectively discounted before estimating heading or path (Adiv, 1985). We follow this idea and study five segmentation cues for IMOs, most of which also account for the case of stationary objects in front of the background.

### Segmentation cues are strong at depth discontinuities introduced by objects and in most cases are altered by more than 30% if these objects move independently from the background

Our aim is to show that segmentation cues for IMOs are different from those at depth discontinuities in most cases, which should allow the observer to distinguish between IMOs and depth discontinuities. We study each of the segmentation cues for (1) only a depth discontinuity or a static discontinuity and (2) a depth and motion discontinuity or dynamic discontinuity (IMO).

#### Segmentation cues for linear motion toward fronto-parallel planes

The depth discontinuity is defined by assigning the distance *d*_0_ to the background and the distance *d*_0_−Δ*d* to the object. For translational motion toward fronto-parallel planes, we assume that the object motion is defined by adding (Δ*v*_*x*_, Δ*v*_*y*_, Δ*v*_*z*_) to the background motion (*v*_*x*_, *v*_*y*_, *v*_*z*_). A dynamic discontinuity introduces the following accretion/deletion:

(27) $$\begin{array}{l} \;\Delta ={\left(-\frac{\Delta d}{(d_{0}-\Delta d)\;\cdot \;d_{0}}\vec{a}({\vec{v}}_{B})+\frac{1}{d_{0}}\vec{a}(\Delta \vec{v})\right)}^{t}{\vec{n}}_{\beta }\text{ with}\\ \vec{a}(\vec{v})=\left(\begin{array}{c} -f\;\cdot \;v_{x}+x\;\cdot \;v_{z}\\ -f\;\cdot \;v_{y}+y\;\cdot \;v_{z} \end{array}\right)\text{ and}\\ \;{\vec{n}}_{\beta }^{t}=(\cos\beta ,\;\sin\beta ). \end{array}$$

Note that the 2nd term in Δ of Equation (27) is introduced by the IMO and, thus, can increase or decrease this cue, which depends on the signs of the two terms in Δ. In the case that $\vec{a}({\vec{v}}_{B})$ and $\vec{a}(\Delta \vec{v})$ fall into different half-planes defined by the normal $\vec{n}$ and Δ*d* < *d*_0_, then the cue is enhanced. For most situations Δ*d* < *d*_0_ holds since the relevant object appears in front of the observer as opposed to Δ*d* > *d*_0_, where it appears behind the observer. In general, it is important that the cue difference between static and dynamic depth discontinuity is strong. To give a qualitative answer we evaluated this difference for β = 0°, *x*, *y* ϵ {−5, −2.5, 0, 2.5, 5}mm, *v*_*xB*_, *v*_*yB*_ϵ {−15, −10, −5, 0, 5, 10, 15}°, Δ*v*_*x*_, Δ*v*_*y*_ ϵ {−5, −3, −1, 1, 3, 5}°, *d*_0_ϵ {5, 10, 15, 20, 25, 30} m. We combine this distance *d*_0_ with Δ*d* being 0.5, 0.75, or 0.875 of *d*_0_. All other parameters are set as in the experiment of Warren and Saunders (1995). We compute Δ for a static depth discontinuity choosing the background motion $\Delta (\Delta \vec{v}=0)$ and count a difference if $\left|\Delta (\Delta \vec{v}=0)-\Delta (\Delta \vec{v})\right|$ is above 30% of $\left|\Delta (\Delta \vec{v}=0)\right|$, the condition for a static depth discontinuity. This criterion is largely based on speed differences between object and background motion which was attributed importance in the segmentation of IMOs (Royden and Moore, 2012). Following this criteria, 63% of all evaluated cases show a 30% difference between static and dynamic discontinuity (IMO).

For expansion/contraction cues we use the same definitions for the distance of IMO and background, and their motions. This results in the cue:

(28) $$\delta =\frac{2}{d_{0}-\Delta d}\left(\frac{\Delta d}{d_{0}}v_{z,\;B}+\Delta v_{z}\right).$$

The IMO motion introduces the 2nd term in Equation (28). Most times humans go forward (*v*_*z*_ > 0). It holds that Δ*v*_*z*_ > 0, if the IMO approaches the observer faster than the background, which is the case in the Warren and Saunders (1995) experiment. Our quantitative analysis gives a 30% difference between a static and dynamic discontinuity (IMO) in 83% of all tested cases.

The acceleration and deceleration cue for the same scene and motion configuration is given by:

(29) $$\alpha ={\left(\frac{2d_{0}-\Delta d}{{\left(d_{0}-\Delta d\right)}^{2}}\frac{\Delta d}{d_{0}^{2}}v_{z,\;B}\vec{a}({\vec{v}}_{B})+\frac{\Delta v_{z}}{{\left(d_{0}-\Delta d\right)}^{2}}\vec{a}(\Delta \vec{v})\right)}^{t}{\vec{n}}_{\beta },$$

where $\vec{a}(\vec{v})$ and ${\vec{n}}_{\beta }$ are defined as in Equation (27). The difference in motion $\Delta \vec{v}$ between background and IMO introduces the 2nd term in Equation (28). Whether this motion difference introduces an increase or decrease depends on the signs of each term. Since there are many cases, we restrict the description to a qualitative analysis with the previously described parameters. Eighty-three percent of all evaluated cases show a thirty percentage difference between a static and dynamic discontinuity (IMO).

The curvature cues for the translational motion are zero-valued in all cases, regardless of the scene geometry, see Table 5.

#### Segmentation cues for curvilinear motion above a ground plane

As next configuration, we study curvilinear motion defined by the linear velocity *v* = ω·*r* and the yaw-rotational velocity ω. The scene is defined as one fronto-parallel plane at the distance *d*_*I*_, which is the IMO, and one ground-plane, the background. The depth discontinuity is expressed in the same way as before; however, the motion for the object ω + Δω and *r* + Δ*r* is now defined using the components Δ*r* and Δω. For a depth discontinuity the accretion/deletion cue evaluates to:

(30) $$\begin{array}{l} \Delta ={\left(\frac{fh-d_{I}y}{d_{I}\;\cdot \;fh}\;\cdot \;\omega \;\cdot \;r\;\cdot \;\vec{x}+\frac{\omega \;\cdot \;\Delta r+\Delta \omega \;\cdot \;r+\Delta \omega \;\cdot \;\Delta r}{d_{I}}\vec{x}-\frac{\Delta \omega }{f}\vec{b}\right)}^{t}{\vec{n}}_{\beta }\\ \text{ with }{\vec{x}}^{t}=(x,\;y),\;{\vec{b}}^{t}=(f^{2}+x^{2},\;xy),\\ \text{ and }{\vec{n}}_{\beta }^{t}=(\cos\beta ,\;\sin\beta ). \end{array}$$

The motion of the IMO introduces the 2nd and 3rd term for Δ. Due to the various signed terms involved, we chose a quantitative analysis rather than denoting all cases separately. For our quantitative analysis we vary parameters in the following ranges: Δ*r* ϵ {−10, −8, −5, −1, 1, 5, 8, 10} m, Δω ϵ {−0.2, −0.15, −0.1, −0.05, 0.05, 1, 0.15, 0.2}rad/s, *v* ϵ {−15, −14, −13, −12, −10, 10, 12, 13, 14, 15} m/s, *r* ϵ {80, 120, 160, 320} m. All other parameters are kept the same as in the experiment of Fajen and Kim (2002). For this setting 91% of all evaluated cases provide a difference above 30% between static and dynamic discontinuity (IMO) taking the static discontinuity as reference.

For the next cue we analyze expansion/contraction for the same configuration. This cue evaluates to:

(31) $$\delta =\omega \;\cdot \;r\;\cdot \;\frac{12fh-3d_{I}y}{d_{I}fh}+2\frac{\omega \;\cdot \;\Delta r+\Delta \omega \;\cdot \;r+\Delta \omega \;\cdot \;\Delta r}{d_{I}}-\frac{3x}{f}\Delta \omega .$$

Note that the motion discontinuity adds the terms Δ*r* and Δω. The additional rotational velocity Δω introduces a dependency on the horizontal position in the image plane. Our quantitative analysis yielded a 30% difference between static and dynamic discontinuity in 89% of all cases.

Next, we derive the acceleration/deceleration cue for curvilinear motion keeping the configuration of scene and motion the same as before. This cue evaluates to:

(32) $$\alpha =\frac{xd_{I}+(r+\Delta r)f}{fd_{I}^{2}}{\left(\omega +\Delta \omega \right)}^{2}\left(r+\Delta r\right)\;\cdot \;{\vec{x}}^{t}{\vec{n}}_{\beta }.$$

Note, that the ground-plane has a zero-valued temporal derivative. Thus, a strong difference occurs due to the varying motion for the condition with and without IMO. This strong difference is also reflected in a 98% occurrence of a 30% difference.

The cues for local spatial curvature and local temporal curvature are summarized without giving the explicit equations. For local, spatial curvature cues a 30% difference appears in 20% of all cases and for local, temporal curvature cues such difference occurs in 21% of all cases. Due to the absence of temporal and spatial curvature cues in the translational motion case and no strong differences for spatial and temporal curvature between static and dynamic objects (IMOs), we excluded these two cues from the further analysis.

### Contour segmentation cues for IMOs are defined along the transition edge between IMO and background

**Figure 4:**
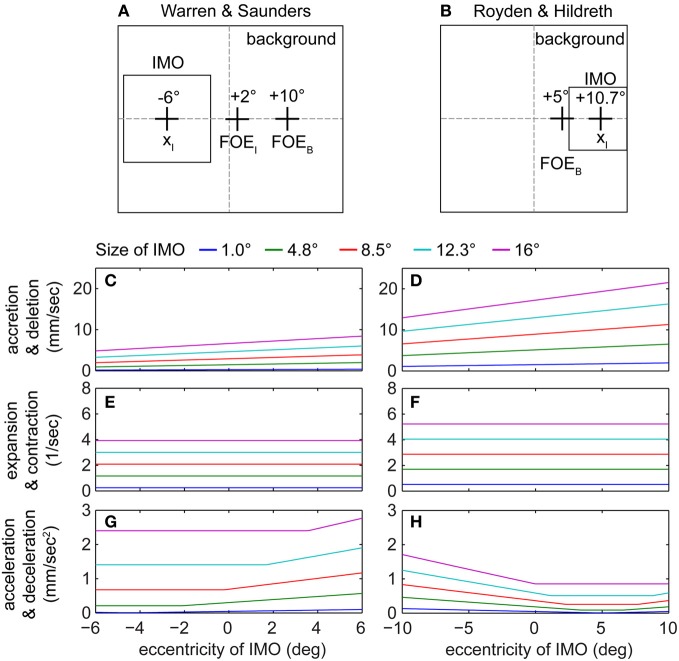
**Shows graphs for the evaluated line integrals of segmentation cue strengths along the contour of the IMO.** Due to the difference in the graph's shape depending on the IMOs horizontal position, these cues form a “basis set” to selectively down-regulate the heading bias or miss-perception in the path for the model. **(A)** One example of the Warren and Saunders (1995) experiments. **(B)** One example of the Royden and Hildreth (1996) experiments. **(C,D)** show the response for accretion and deletion, **(E,F)** for expansion and contraction, and **(G,H)** for acceleration and deceleration for the two examples from **(A,B)**, respectively.

Figure 4 shows the evaluation of these contour segmentation cues for different sized IMOs. We kept parameters the same as described before and additional parameters are set as follows: β = 45°, *v*_*x*, *B*_ = 10°, *v*_*y*, *B*_ = 0°, *v*_*x*, *I*_ = 2°, and *v*_*y*, *I*_ = 0°. Figure 4A shows one exemplar configuration from the Warren and Saunders (1995) set of experiments, and Figure 4B shows one exemplar configuration from the Royden and Hildreth (1996) set of experiments for: *v*_*x*, *B*_ = 5°, *v*_*y*, *B*_ = 0°, and β = 45°. Figures 4C,D show accretion/deletion cue strengths for these two examples when varying the horizontal position of the IMO and its size. In both cases the trend of the data from the simulation is the same with strong cues being present on the right-hand side of the visual field where the FOE of the background is located. This is explained by the definition of our measure for contour segmentation. Accretion/deletion is measured normal to the edge, and we compute the sum of absolute values taken for each line integral [compare with Equation (25) and Figure 1D]. Cues for the upper and lower edge of the IMO are strongest if this IMO is located close to the FOE of the background, which explains the trend given in the data which graphs of Figures 4C,D show. These cues increase with the size of the IMO as well. The accretion/deletion cue in the Royden and Hildreth (1996) example is stronger than in the Warren and Saunders (1995) example. Figures 4E,F show expansion and contraction cues. These cues are not only independent of the horizontal or vertical position of the FOE but also independent of the spatial location of the IMO, see Table 5. Figures 4G,H show acceleration/deceleration for the two experiments. For this case the trend in the curves is different between the two examples. In Figure 4G the strength of the cue increases the further the IMO is shifted toward the right side of the visual field, toward the FOE of the background motion. When IMO and FOE overlap, vectors of the IMO and the background point approximately in the same direction while their lengths differ. Lengths of vectors that belong to the IMO are longer than those from the background. The length of vectors from the IMO scales by 1/*d*^2^_0_. In comparison, the length of vectors from the background scales by 1/*d*_0_. For the Royden and Hildreth (1996) example, which Figure 4H shows, the trend is a U-shaped curve. When the IMO overlaps with the position of the background FOE the acceleration/deceleration is weakest. In this example, all flow vectors of the IMO point to the left. Thus, if the IMO is located on the right-hand side of the visual field then this cue has a small magnitude, since background vectors and IMO vectors point largely into the same direction. In contrast, if the IMO appears on the left-hand side of the visual field, vectors of IMO and background point into opposite directions, leading to a strong acceleration/deceleration magnitudes.

To fit human heading biases, which are large for a crossing IMO, in the model we mainly use acceleration/deceleration as a segmentation cue, since it has the appropriate characteristics for reducing the heading bias in the correct conditions: acceleration/deceleration is strong when the IMO's position is far from the background's FOE, which is the non-crossing case. In this case the cue can effectively reduce the bias term. When the IMO's position is close to the background's FOE the acceleration/deceleration is weak and, thus, the bias largely remains. The next subsection will discuss this idea in more detail and provides data fits.

### Including segmentation cues into a linear least square estimation explains measured heading biases and curvilinear path perception

**Figure 5:**
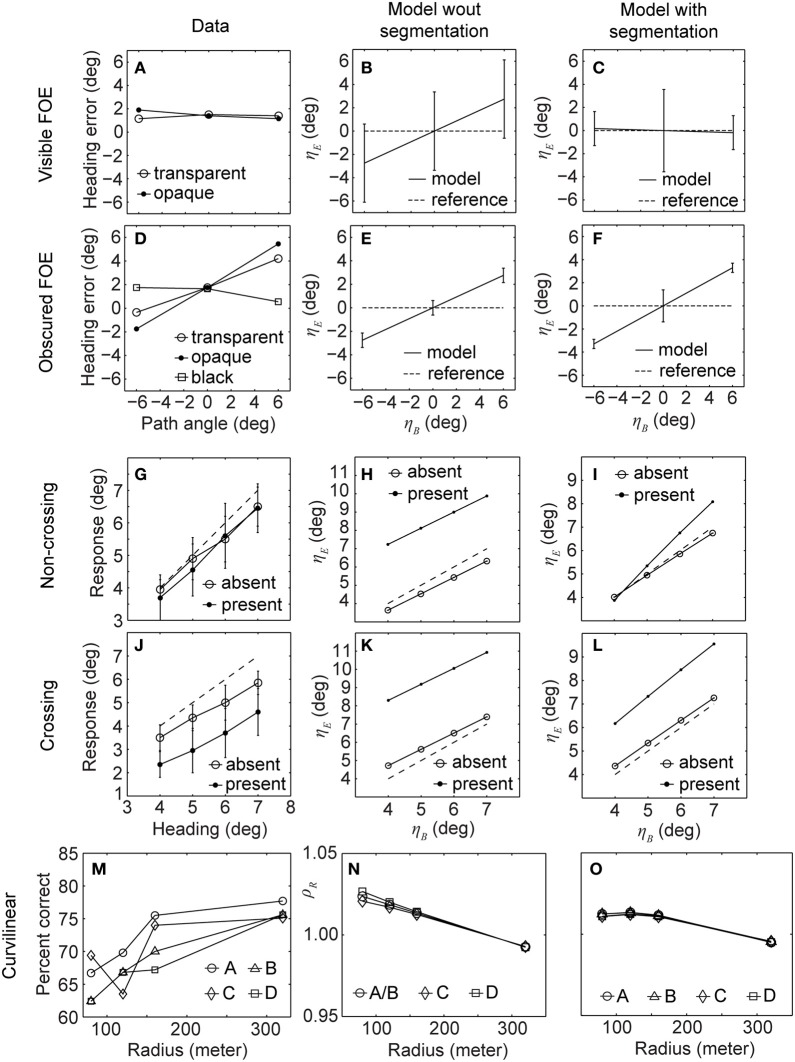
**Shows data from the original experiments (1st column) and our model without (2nd column) and with segmentation (3rd column).** While the model without segmentation did not fit the data very well, the model with segmentation does so for all three sets of experiments (Warren and Saunders, 1995; Royden and Hildreth, 1996; Fajen and Kim, 2002). **(A,B,C)** Show the heading error for humans, our model without and with segmentation, respectively, when the FOE is visible. **(D,E,F)** Show the heading error for the same three categories for an obscured FOE. **(G,H,I)** Show estimated heading for humans, our model without and with segmentation, respectively, when the IMO's path and observer's path are not crossing. **(J,K,L)** Show the estimated heading for the same three categories for crossing paths. **(M)** Percent correct responses of humans estimating future path for four different configurations labeled as A, B, C, and D. **(N,O)** Error of the radius estimated by our model for the same configurations without and with segmentation, respectively.

To fit the data on heading biases and path perception, we use accretion/deletion, expansion/contraction, and acceleration/deceleration cues. These three cues have different graphs depending on the horizontal position of the IMO. Some graphs have zero slope, others have a left/rightward slope, and yet others have multiple slopes. This difference in characteristics enables us to use them as a “basis set” of components to selectively down-modulate heading biases. Our aim was not to find the best fit, e.g., lowest *r*^2^-error; rather we fit the curves by hand, changing the weights of these three segmentation cues using a weighted linear combination (see section Methods). Figure 5 shows the graphs for heading bias and path perception using existing psychophysical data for the fit (Warren and Saunders, 1995; Royden and Hildreth, 1996; Fajen and Kim, 2002). Parameters for the experiment settings were kept the same as described for Figure 3. We show results from Figure 3 to allow for a better comparison between the data, our model without segmentation cues, and our model with segmentation cues. Figures 5A–F shows the heading bias for the conditions of visible (first row) and obscured FOE (second row). The data fit for Figures 5A–F has the parameters: *c*_Δ_ = 10^5^, *c*_δ_ = −6 × 10^2^, and *c*_α_ = 4.5 × 10^5^ [compare with Equation (26)]. Including the segmentation cue into our model allows us to account for the perceptual observations and, thus, the fitting of behavioral data: for the condition of a visible FOE, the mean values for heading biases range all around zero while providing a flat graph that fits better to the characteristics of data, as seen in Figure 5C. Without segmentation the graph showing the simulation results has a positive slope, see Figure 5B. For the obscured FOE, including the segmentation cue into the model does not change the heading biases substantially (compare Figures 5E,F). However, the standard deviations are slightly reduced for the model with segmentation compared to the model without segmentation, which would make a significance test against a zero bias stronger. Note that both models reflect the data plotted in Figure 5D. The segmentation cue could not generate the bias of estimated heading toward the center of the screen as visible in the data, see Figures 5A,D.

Figures 5G–L show results for the next set of experiments, the 3rd row for a non-crossing IMO and the 4th row for a crossing IMO. Parameters of the fit are: *c*_Δ_ = 3.5 × 10^5^, *c*_δ_ = −10.5 × 10^2^, and *c*_α_ = 3 × 10^5^. In this case the segmentation cue helps to improve the results and reduce the deviation between ground-truth horizontal heading (diagonal in the graphs) and estimated horizontal heading. Data from Royden and Hildreth (1996) is plotted in Figures 5G,J for non-crossing and crossing IMO, respectively. Model results without segmentation produced large deviations as visible by comparison of the graphs in Figures 5G,J with those in Figures 5H,K. Note that the panels use different scales. Including the segmentation cue into the model helped to reduce this deviation, in particular in the non-crossing IMO case, see Figure 5I. For the crossing case the model with segmentation overestimates the heading by about 2° independent of the heading; this is similar to the data in terms of the strength of deviation. Data from the human subject shown in Figure 5J underestimated heading by about 2°. The difference in bias direction is not critical as Royden and Hildreth (1996) point out that the direction of deviation was not consistent across subjects.

Figures 5M–O shows the estimation of curvilinear path for data, the model without segmentation, and the model with segmentation, respectively. Parameters of the fit are: *c*_Δ_ = 10^4^, *c*_δ_ = 10^1^, and *c*_α_ = 10^4^. The first two panels were described along with Figure 3. Including the segmentation cues shifts the data points for the radius *r* = 320 m closer to a unit ratio and, furthermore, reduces the deviation for smaller radii; see Figure 5O. Note that the trend, smaller error for high curvatures, is maintained when incorporating the segmentation cue into the model. The important observation is that various cues of segmentation can be viewed as forming a “basis set” of the necessary constituencies to be linearly combined in order to selectively reduce deviations for configurations in which either the IMO's path is crossing the observer's path or the IMO is obscuring the position of the FOE from the background motion.

## Discussion

We derived expressions for the heading bias and radius estimate for linear motion and curvilinear motion as well as five segmentation cues. These cues are accretion/deletion, expansion/contraction, acceleration/deceleration, spatial, and temporal curvature. Such cues and their definition are general enough to be studied for real-world video, e.g., in combination with existing bio-inspired flow detection models (Pauwels et al., 2010), see also Figures 1C–G. A linear method of flow-based estimation for heading or path radius could not explain the observed human biases. Humans show heading biases if the IMO covers the FOE of the background motion or the IMO's path intersects with the observer's path (Warren and Saunders, 1995; Royden and Hildreth, 1996). However, humans accurately predict future path for curvilinear motion in the presence of IMOs. These seemingly contradictory results might be explained by segmentation cues utilized by the visual system to improve the judgments about heading or path as these cues differ between experimental conditions. In studies that probed heading, fewer segmentation cues are available in theory than in those studies that probed future path. In particular, local spatial and temporal curvature cues are absent for linear motion used to probe heading. Our linear method for the estimation of heading or path radius improved when integrating segmentation cues. This improvement is expressed in a better fit to data from psychophysical experiments. We use different weights for the segmentation signals in each study, which we do not view as critical since motion and scene parameters differed widely between these studies. Our computational study suggests that segmentation cues play a role in the perception of heading and path when IMOs are present. Those cues are used to reduce or discard the IMO's influence for motion integration. We discuss this suggestion in the context of recent studies on heading and path perception that present alternative views.

### Conceptual and computational models for the segmentation of IMOs and estimation of self-motion

An alternative suggestion to our flow segmentation is flow parsing, the segmentation of IMOs employing global cues rather than a local flow segmentation that we proposed. Warren and Rushton (2009a) provide evidence that humans use such flow parsing strategy in which the majority of the flow is consistent with self-motion through a hallway. The IMO appears as a circular disk falling to the ground. The proposal is that humans employ a three-step procedure to recover all motions in the stimulus. First, the visual system accesses the self-motion based on dominating flow patterns from the background. Second, the globally predicted flow for this self-motion is subtracted from the sensed flow. Third, the residual motion is the motion of the IMO that can be estimated. Aside from semantic cues (falling ball) being present and choosing a parameterization where no heading bias was reported before (compare Warren and Saunders, 1995; Royden and Hildreth, 1996), Warren and Rushton do not explain how the scene is initially segmented to access self-motion from flow originating from the background. In a recent study Warren et al. (2012) point out that flow parsing might not depend on prior heading estimation. In contrast, our study provides a specification of the mechanisms for such an initial segmentation that could be used to estimate self-motion from the background flow mainly. The features and properties are defined on the basis of monocular depth cues that could be enhanced even further using available binocular depth cues (Warren and Rushton, 2009b).

An alternative hypothesis suggests combining segmentation and self-motion estimation. Hildreth (1992) and Royden (2002) proposed a computational model that employs motion-opponent operators. These operators locally compute the motion difference and only strong differences are further considered. A voting strategy with these motion differences yields the translational self-motion velocity assuming that the background subsumes the largest area in the image. The motion-opponent computation segments linear from rotational velocity (Rieger and Lawton, 1985) and the voting mechanism segments self-motion from IMOs. Due to voting biases occur in Royden's model when background and IMO motion are similar, e.g., when their paths cross. Our model explains the data (Royden and Hildreth, 1996) and extends it by providing an explanation for curvilinear path data (Kim, 2000; Fajen and Kim, 2002).

Adiv (1985) suggests a similar computational approach that uses the optic flow Equation (1) for plane models. Assuming that the scene can be approximated by local planar patches, all parameters—linear motion, rotational motion, and those of the plane—are constant within such a local region. A voting (grouping) mechanism for such parameters that are locally constant can indicate an IMO who will disagree due its motion difference.

Layton et al. (2012) suggest a partly recurrent neural network of motion integration mechanisms that fits data of heading biases for linear path perception; however, it remains unclear if the same model mechanisms could account for the data of curvilinear path perception.

Pauwels and Van Hulle (2004) propose an iterative mechanism to combine segmentation and estimation of self-motion. The first step estimates the self-motion parameters by employing a weighted, adaptively de-biased bilinear constraint (Bruss and Horn, 1983). The second step computes a residual translational flow by subtracting the estimated analytical rotational flow from the input flow. This residual translational flow is compared to the analytical translational flow based on the angular difference between individual flow vectors. This difference is large for regions that contain IMOs and small for the background, and is used to define weights. These weights are the “glue” between first and second step of the iterative optimization process and segment IMOs from the background.

Other methods solve segmentation and heading estimation with the expectation and maximization (EM) algorithm (MacLean et al., 1994; Clauss et al., 2005). The expectation step estimates the probability of a data point as being generated by a motion model. Maximization is achieved by formulating a probability weighted least squares problem for the estimation of self-motion or object-motion from optic flow. Both steps are iterated until convergence is reached. In practice, this convergence is assumed when the change in probability assignments between successive iterations is below a threshold.

Another strategy to solve the intertwined problem of segmentation and motion estimation uses the random sample consensus (RANSAC) method (Fischler and Bolles, 1981). This method iteratively finds a set of data points, which is consistent with the self-motion model assuming that the background subsumes the largest area in the image (Raudies and Neumann, 2012). All these approaches do not require a prior segmentation as suggested in our work; however, they could profit by it. Segmentation could be included as a prior for the voting methods and as an initialization for the iterative methods.

### Steering control

Our analytical models have implications for steering control. Cutting et al. (1995) suggest that stationary obstacles are avoided by using motion parallax while keeping fixation on the object off to one side. We used similar motion models to qualitatively evaluate the scenario of avoiding a stationary obstacle (Raudies et al., 2012). Here, we further refined these models to account for IMOs, which shows that segmentation cues of moving obstacles are enhanced in most cases compared to stationary ones. Fajen and Warren provided data and a dynamical force model for the avoidance of stationary/moving obstacles and the pursuit of a moving target (Fajen and Warren, 2003, 2004; Warren and Fajen, 2008). Their model uses a birds-eye view perspective, assuming knowledge about the position of self and obstacles in polar coordinates. Goals act as attractors and obstacles as repellers in a 2nd-order dynamical system.

Other models take a first-person perspective approach for steering control (Browning et al., 2009; Elder et al., 2009). These models use analytical flow or detected optic flow to estimate the horizontal position of objects and heading. The estimated object positions are fed into a control circuit where obstacles are represented by negative Gaussians attached to their horizontal position in the visual field, the target by a positive Gaussian represented at its horizontal position, and the horizontal heading as another positive Gaussian. Superimposed entries from all horizontal positions are summed and the resulting distribution defines the steering variable for the yaw velocity.

Our study differs by using an analytical approach. The derived segmentation cues extend the overall functionality of models that have been previously proposed. In addition to deriving these cues, we incorporate them into a mechanism that selectively integrates motion in order to estimate heading or path. This first-person perspective also accounts for the size of target or goal object in the visual field. Such an approach could implicitly account for the distance dependence in the dynamics due to the effect of looming that is present for the first-person perspective (Fajen and Warren, 2003).

### Path perception

In our analysis heading was tangent to the path; however, in general, this can differ. Instantaneous flow is ambiguous with respect to eye-rotations and or body/head-rotations. Given eye-rotations and a straight path trajectory, flows have a rotational component. For body-rotations the path trajectory is curvilinear and the flow has a rotational component too. The ambiguity in the instantaneous motion field yields to a strong bias with respect to the expectation about the path either being linear or curved as given, e.g., by the instruction as part of the experiment (Li and Warren, 2004). Only when gaze pointed along the heading direction (zero heading), path perception is accurate (Li and Cheng, 2011). The perception of curvilinear paths is improved by dense motion parallax cues and a reference object (Li and Warren, 2000). In our formulations for curvilinear path motion gaze and heading were aligned, which resolves the ambiguity. Several degrees of freedom e.g., eye-movements (Warren and Hannon, 1990; Royden et al., 1992, 1994), have been left out in our model to focus on data from Warren and Saunders (1995), Royden and Hildreth (1996), and Fajen and Kim (2002) and not to further complicate the model equations. Future work will focus on the interplay between eye-, head-, and body-movements during linear or curvilinear path motion and the various segmentation cues present in the generated flows.

### Conflict of interest statement

The authors declare that the research was conducted in the absence of any commercial or financial relationships that could be construed as a potential conflict of interest.
